# Cardiomyopathy in Sickle Cell Disease

**DOI:** 10.7759/cureus.9619

**Published:** 2020-08-08

**Authors:** Harsimran Kaur, Fahad Aurif, Mahdi Kittaneh, Jeoffrey Patrick G Chio, Bilal Haider Malik

**Affiliations:** 1 Internal Medicine, California Institute of Behavioural Neurosciences and Psychology, Fairfield, USA; 2 General Surgery, California Institute of Behavioural Neurosciences and Psychology, Fairfield, USA; 3 General and Laparoscopic Surgery, California Institute of Behavioural Neurosciences and Psychology, Fairfield, USA; 4 Family Medicine, California Institute of Behavioural Neurosciences and Psychology, Fairfield, USA

**Keywords:** cardiomyopathy, restrictive cardiomyopathy, iron overload cardiomyopathy, sickle cell disease, chronic anemia, microvascular occlusion, diastolic dysfunction, pulmonary hypertenion

## Abstract

Sickle cell disease (SCD) is an inherited disorder that occurs due to point mutation in the beta-globin chain resulting in the production of hemoglobin S that tends to become rigid and sickle-shaped under low oxygen concentration. These sickle-shaped red blood cells (RBCs) obstruct the blood vessels leading to reduced blood flow to the organs, causing ischemia and tissue fibrosis. These sickle RBCs being abnormal in shape are frequently sequestered by the spleen, creating a state of chronic anemia in the body. This chronic anemia leads to a high cardiac output state causing cardiac remodeling. To tackle chronic anemia, patients are frequently treated with blood transfusions that makes them more prone to the risk of iron overload (from newly transfused RBCs and iron release from the RBCs that just got sequestered as well as from volume overload) and volume overload causing left ventricular (LV) dilation. The above-mentioned mechanism of cardiac hypertrophy, along with LV dilation together, makes SCD-related cardiomyopathy unique cardiomyopathy with features of restrictive cardiomyopathy with LV dilation. It is interesting to note here that even though there is a presence of LV dilatation, Systolic dysfunction is very uncommon in SCD-related cardiomyopathy.

## Introduction and background

Complications of sickle cell disease (SCD) represent the significant health care burden in developed countries and cardiovascular (CV) complications being the highest causes of mortality [1]. SCD is an inherited blood disorder in which point mutation in the beta-globin gene results in the production of hemoglobin S that polymerizes within the erythrocyte during deoxygenation, making red blood cells (RBCs) rigid and sickle-shaped. This hemoglobin S creates a state of chronic hemolytic anemia due to splenic sequestration of abnormal erythrocytes. Chronic anemia in SCD results in prolonged exposure to a high cardiac output state that causes compensatory eccentric myocardial remodeling, resulting in left ventricular (LV) hypertrophy, dilation, and atrial distension [1]. This chronic anemia is treated with frequent blood transfusions, which in turn leads to iron overload. Excess iron (accumulated from splenic sequestration and chronic blood transfusions) is released into the circulation, causing saturation of the carrying capacity of transferrin, and non-transferrin bound iron (NTBI) will appear in the serum [2]. This free iron is taken up by cells of various organs, including heart that increases reactive oxygen species (ROS) production and causes cellular damage, mitochondria being the major site of iron metabolism and ROS production [3]. Heart damage occurring due to the above-mentioned mechanism causes unique cardiomyopathy, that has the features of restrictive cardiomyopathy with defining features as diastolic dysfunction, LA enlargement, and normal systolic function along with LV enlargement. The LV dilation occurring in SCD does not fit into the standard definition of restrictive cardiomyopathy but occurs due to the additional presence of the hyperdynamic state in the body [4]. The effects of SCD on the heart is very well known. Here we aim to study the pathophysiology of cardiomyopathy occurring in SCD, that is, how does chronic anemia affect the body, how does chronic blood transfusions help SCD patients, and if chronic blood transfusions cause iron overload in the body. Also, if restrictive cardiomyopathy is caused by iron overload, it raises a question of whether frequent transfusions are causing more harm than good, which will lead us to look for the factors that protect the myocytes from iron load in SCD patients. In addition to this, we will also study how sickle RBCs cause vaso-occlusion in the heart. With all these questions in mind, we will be searching for literature on PubMed and will write a review article in which we will try to answer these questions. This will help to educate people suffering from SCD and will help physicians to update guidelines for the treatment plans of SCD, which will ultimately improve the quality of life of the patients.

## Review

Chronic inflammation in SCD

**Figure 1:**
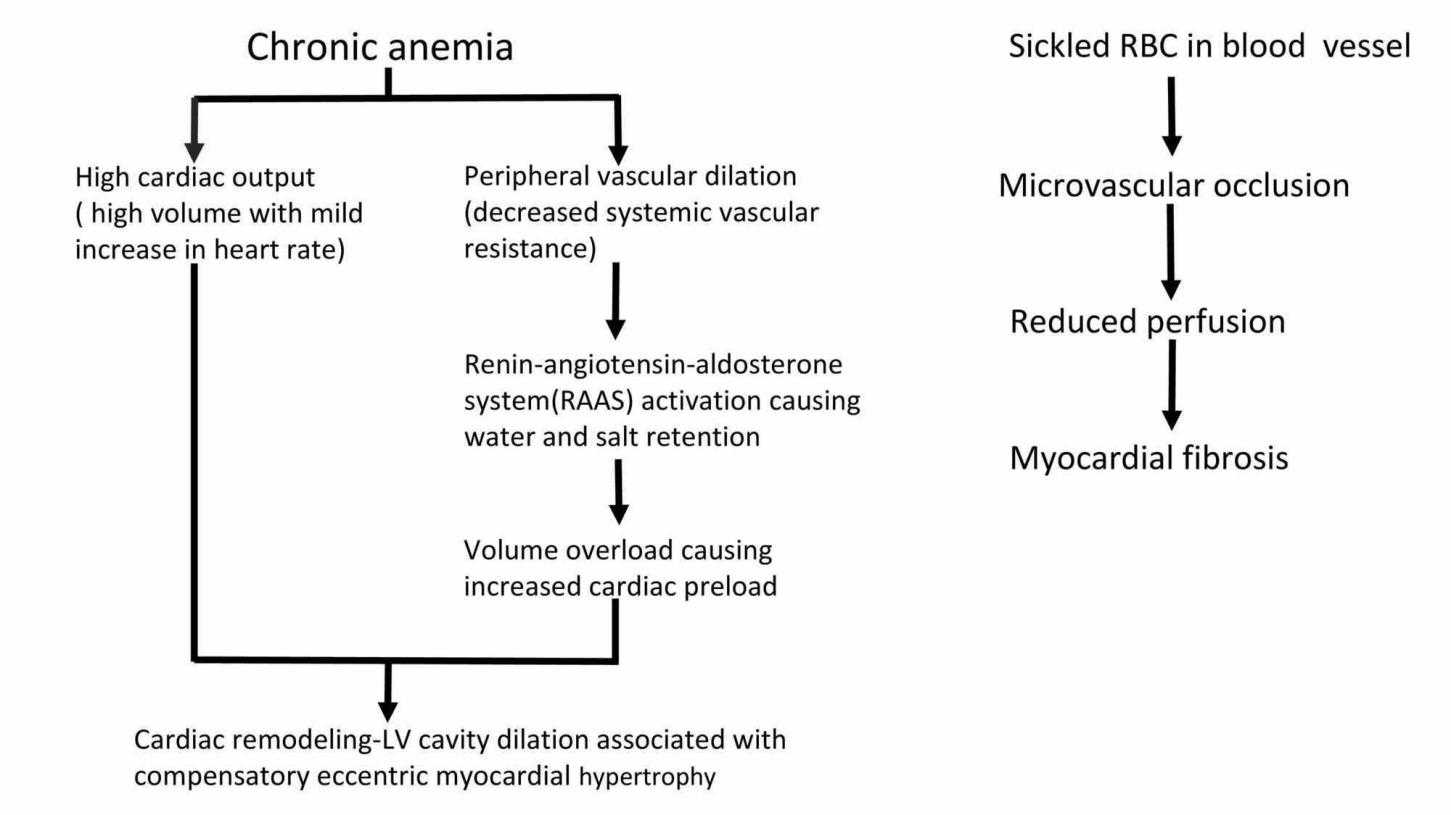
Sequential changes in pathophysiology of SCD-related cardiomyopathy

Chronic inflammation has been said to play an essential role in the pathogenesis of SCD, reactive oxygen species (ROS) generated by leukocytes, endothelial cells, plasma enzymes, and sickle red blood cells being its important component [5]. Human and animal models identified elevated TNF-α, IL-6, IL-10, and hsCRP as markers of inflammation [6]. Xanthine oxidase released from the liver, nitric oxide and secondary oxides of nitrogen and endothelial NADPH oxidase has been the contributing source of oxidants in SCD [5]. ROS generation in SCD has been attributed to sickle hemoglobin, which binds to the RBCs and acts as a reagent in Fenton chemistry reaction leading to generations of superoxide and hydroxyl radicle [5]. Increased fragility and rigidity of RBC in SCD caused by ROS, further acts as a positive feedback to pathophysiologic findings in SCD, including hemolysis and vaso-occlusion (Figure 1) [5]. This chronic inflammation does not just cause damage; instead, it has a unique physiology that protects organ injury by keep NTBI restricted within the reticuloendothelial system and maintaining protective antioxidants in the body [6].

Pathophysiology of SCD-related cardiomyopathy and its complications

SCD, which originated from Sub-Saharan Africa, Southeast Asia and India, has been known to be the most common inherited hemoglobinopathy worldwide due to increasing migration in the modern world. Prolonged exposure to high cardiac output state from chronic anemia in SCD leads to significant dilatation of LV which, over time, adapts to increased wall stress by developing eccentric LV hypertrophy, as shown in Figure 1 [1,7,8]. Myocardial remodeling can be well tolerated for a long time; however, myocardial damage can occur from chronic volume overload leading to exercise intolerance and heart failure. In the recent study, the pattern of heart failure in Afro-Americans (AA) with SCD versus heart failure in AA without SCD was studied with the help of the National Inpatient Sample Database (NIS), which represents more than 97% of the US population. While it is known that heart failure occurs at an earlier age in African-American (AA), but this study observed that the chances of getting heart failure increase even further in AA patients with SCD [1].

Myocardial fibrosis in SCD

Intravascular hemolysis causing nitric scavenging, along with repeated vaso-occlusion by sickle RBC can contribute to microvascular dysfunction, which has been identified to cause cardiac dysfunction in SCD patients (Figure 1) [9,10]. Many studies have studied the myocardial function and have shown the presence of myocardial fibrosis along with abnormal perfusion reserves with the help of non-invasive cardiovascular testing [11,12]. These studies tried to identify the potential causative mechanism behind microvascular dysfunction, and they successfully ruled out myocardial iron overload in those patients, reducing the possibility of iron overload causing restrictive cardiomyopathy in SCD patients [11,12]. The first study chose five asymptomatic SCD patients (1M/4F), median age: 28 (range: 17-33), to evaluate their myocardial function, perfusion reserve, fibrosis and iron overload using cardiovascular magnetic resonance (CMR). Four out of five patients showed myocardial ischemia. Diffuse myocardial fibrosis was seen in all the patients, no patient had myocardial iron on heart T* but liver T* showed significant iron overload [12]. This study concluded that repeated vaso-occlusive episodes, reduced vascular bed by impaired vaso-reactivity and reperfusion-induced injury is the crucial pathological mechanism behind their findings. In the second study, 38 stable SCD black patients were studied and were compared with 13 healthy controls; controls were frequency-matched on age, sex, and race with SCD patients. Patients had 21% reduced myocardial perfusion than healthy controls along with the presence of myocardial fibrosis in 25% of the patients, and only one patient had the evidence of myocardial iron on the heart [11]. The study associated LV dilation and myocardial fibrosis with increased blood transfusion requirements. Diastolic dysfunction was correlated to aortic stiffness and after-load, which was seen mainly in older age, increased blood pressure and high creatinine in SCD [11].

Myocardial iron overload uncommon in SCD

Chronic blood transfusion-related iron overload cardiomyopathy is a major cause of death in patients with chronic anemia, such as in thalassemia or SCD [2]. High mortality is caused by increased ROS generated by high iron present inside the mitochondria of the cell [2,3]. Iron overload can cause iron overload cardiomyopathy if excessive iron enters cardiomyocytes. However, many studies were able to prove that iron overload is not a common phenomenon, as seen on the cardiac resonance imaging (CMR) using T2* imaging in patients with SCD [6,13]. Myocardial iron deposition may only occur in 2%-5% of SCD patients with chronic blood transfusions, which may lead to systolic dysfunction, heart failure, arrhythmias, and sudden death in patients when present [14,15]. The first study showed that out of 200 homozygous SCD patients that they studied, only seven had myocardial iron deposits. Out of seven, five of them had ferritin higher than 3000 μg, and two had between 1500 μg and 2000 μg. All of these patients had transferrin saturation above 60% and elevated NT-proBNP (or BNP). All patients had symptoms of acute heart failure. Two out of seven had heart failure with reduced ejection fraction (HFrEF), while five had heart failure with preserved ejection fraction (HFpEF). Iron chelation therapy helped patients with HFrEF in partially recovering their ejection fraction [14]. Similarly, the other study that included 201 SCD patients revealed only five (2.5%) patients showed myocardial iron overload [15]. This is in comparison to chronic transfusion in beta-thalassemia, where cardiac toxicity from myocardial deposition remains the major cause of mortality [15-17]. To explain the reason for low chances of myocardial iron in SCD, a hypothesis was put forward which included later onset of chronic transfusion in sickle cell anemia (SCA) as compared to thalassemia, use of erythrocytapheresis rather than simple transfusion, chronic inflammation that sequesters iron within reticuloendothelial cells and efficient erythropoiesis capable of handling the iron from both transfused blood and hemolysis [15,17].

Diastolic dysfunction in SCD

Many studies identified diastolic dysfunction in SCD as an independent risk factor for premature death [18-20]. Factors that lead to diastolic dysfunction are either intrinsic or extrinsic to the myocardium. While iron overload is an intrinsic myocardial cause and abnormal neurohormone activation, causing abnormal myocardial relaxation along with myocardial hypertrophy from chronic anemia forms the possible intramyocardial causes of diastolic dysfunction [21]. The study enrolled 30 children (median age 13) with SCD with serum ferritin of 1000 ng/ml or more to study diastolic dysfunction in chronically transfused patients. The tissue Doppler echocardiographic findings showed 23 patients with abnormally low mean mitral annular velocity (e′) corresponding LV diastolic dysfunction and 13 patients had abnormally low mean tricuspid annular velocity (e′) corresponding right ventricular diastolic dysfunction, respectively. However, none had abnormally T2*-MRI values indicating that iron overload is not related to diastolic dysfunction in SCA [21]. Another study which included 26 SCD patients (mean age: 23 ± 13 years) to study the association of diffuse myocardial fibrosis to diastolic dysfunction by measuring extracellular volume fraction (ECV) with the help of CMR, found that its ECV was increased in all the patients including young children. It was able to show that myocardial fibrosis was associated with diastolic dysfunction and restrictive pathophysiology of SCA-related cardiomyopathy [18].

Pulmonary hypertension in SCD

Pathophysiology of pulmonary hypertension (PH) in SCD is complex and is a significant cause of mortality and morbidity in patients [22-25]. A study showed that out of 24 confirmed PH patients as diagnosed by right heart catheterization, 11 patients had pre-capillary hypertension, and 13 had post-capillary PH [22]. There are several mechanisms involved in SCD-related PH. First, as pressure is a product of vascular resistance and flow, high cardiac output causes increased pulmonary pressure regardless of whether pulmonary vascular resistance is high or not [26,27]. Second, chronic volume overload subsequently causes high pulmonary venous pressure [22,28]. Third, nitric oxide scavenging due to free plasma hemoglobin can cause intravascular hemolysis, which can induce pulmonary arterial vasculopathy [29]. Out of three mechanisms, two are directly linked to the hyperdynamic state due to chronic anemia. A study conducted on 122 stable patients with SCD disease also demonstrated that it might be the chronic anemia which is the dominant factor responsible for abnormal cardiopulmonary hemodynamics in patients with SCD, echocardiographic evaluation showed 36% of patients had a tricuspid regurgitant velocity ≥2.5 m.s-1; however, only 2% had elevated pulmonary vascular resistance [26]. In SCD, diastolic dysfunction and PH each contribute independently to mortality and patients with both risk factors present have very poor prognosis [19].

Preventive measures to cope with complications

By diluting the load of sickle red blood cells in the circulation, chronic blood transfusion may reduce the risk of vaso-occlusion and stroke [30]. However, it also leads to cardiac remodeling, that is, LV dilation from volume overload. Consequently, this whole process causes myocardial damage leading to complications such as heart failure. To prevent these long-term complications of volume overload, there should be strict guidelines to simultaneously screen and manage volume overload in patients receiving chronic blood transfusions, more emphasis should be given to the patients with high risks to developing heart failure such as AA. This will improve the quality of life of SCD patients. Also, It is not much known if calcium-channel blockers, particularly dihydropyridines such as amlodipine and nifedipine, would help prevent complications such as stroke as they cause peripheral vasodilation. We would highly encourage future researchers to study the effects of calcium channel blockers in SCD patients. Talking about the limitation of this paper, the studies that explained iron overload cardiomyopathy in SCD were conducted on animals, which make them a piece of weak evidence. On the other hand, the studies which helped us to explain how myocardial fibrosis causes cardiomyopathy in SCD patients were conducted on very tiny populations which made us doubt their reliability. Therefore, we encourage future researchers to study the phenomenon of cardiomyopathy on a larger population.

## Conclusions

This study was conducted to demonstrate the pathophysiology of cardiomyopathy occurring in patients with SCD. It aimed to study the cause behind the restrictive nature of SCD-related cardiomyopathy and the impact of chronic anemia on the heart and its long-term complications in patients. It was very interesting to note that the patients who had diastolic dysfunction did not have myocyte iron overload, as confirmed by CMR, which means iron overload was not the cause of restrictive cardiomyopathy in patients. Instead, it occurs due to myocardial fibrosis from vaso-occlusion as well as from ventricular hypertrophy caused by chronic anemia. This restrictive cardiomyopathy, along with LV dilation from the hyperdynamic state in the body together, makes SCD-related cardiomyopathy unique cardiomyopathy. This paper is crucial as it explains the complicated nature of cardiomyopathy related to SCD, which will help clinicians to understand the pathophysiology of SCD-related cardiomyopathy and will help them with treatment plans for patients. It will also help to educate patients about their disease. During our literature search, we realized that even though frequent transfusions are required to treat chronic anemia and to dilute sickle cells in the blood, it has its side effects too. We encourage future researchers to study the consequences of frequent transfusions in order to lay down strict guidelines for its early management and prevent various complications arising from volume overload.
